# Rice body synovitis in pediatrics: three different case reports

**DOI:** 10.3389/fped.2024.1391229

**Published:** 2024-06-13

**Authors:** Liping Wang, Yingying Jin, Hua Huang, Zhen Yang, Fei Ding, Xuemei Xu, Chenxi Liu, Shengfang Bao, Xiqiong Han, Jing Ma, Yanliang Jin, Haiqing Cai

**Affiliations:** ^1^ Department of Rheumatology and Immunology, Shanghai Children’s Medical Center, School of Medicine, Shanghai Jiao Tong University, Shanghai, China; ^2^ Department of Pathology, Shanghai Children’s Medical Center, School of Medicine, Shanghai Jiao Tong University, Shanghai, China; ^3^ Department of Orthopedic, Shanghai Children’s Medical Center, School of Medicine, Shanghai Jiao Tong University, Shanghai, China

**Keywords:** rice body, synovitis, children, imaging, histology

## Abstract

Rice body synovitis (RBS) is a rare disease, especially in children. Rheumatoid disorders and tuberculosis are the first two reasons for the formation of the RB. The diagnosis is mainly based on imaging and histopathological features. Herein, we report three cases of RBS in children diagnosed with congenital synovial chondromatosis, tuberculosis (unconfirmed), and ANA -positive juvenile idiopathic arthritis. Clinical features, radiographic findings, pathophysiology, treatment process, and prognosis were reviewed and documented meticulously to enhance cognition in this population and provide some references for clinicians in diagnosing and treating the disease.

## 1 Introduction

The rice body (RB) is a rice-like body with a hyaline chondroid appearance and is mainly found in the synovial fluid of joints and extra-articular tissues. Hyperplasia of the synoviocytes and thickening of the subintimal layer is the predominant pathological condition in the chronic phase (1). Inflammatory cells, nodular granulomas harboring multinucleated giant cells and capillary hyperplasia and telangiectasia can be found in the subintimal layer with the disease development. RB synovitis (RBS) was first reported by Riese in 1895; the microscopic characteristics of RBS in tuberculous arthritis were described in detail (2). RBS is an unusual lesion; data on its incidence is lacking to date. Notably, the development of RBS in adults is well known; however, it is possibly overlooked and rarely reported in children. In 1979, Wynne-Roberts described the first pediatric case of RB formation in rheumatoid arthritis (3). In his classic study, he reported the microscopic appearance of the cells in the matrix of RBS. Druschel reported two cases of children younger than three years of age with multiple RBS in the knee joint (4). RBS are always found in joints or joint-associated bursas as an accompanying manifestation of some systemic diseases. In addition to tuberculosis and rheumatoid diseases, many other diseases are associated with RBS. Identification of additional synovitis diseases is necessary before diagnosis so that patients can be treated based on etiology. In the present study, we summarize the clinical similarities and differences of RBS between three pediatric cases with different causes, including congenital synovial chondromatosis, unconfirmed tuberculosis, and ANA-positive juvenile idiopathic arthritis (JIA). We hope that these documents can enhance understanding and provide a reference for the diagnosis and treatment of RBS in children.

## 2 Cases description

Here, we report RBS in three pediatric cases, whose clinical details are presented in Table 1. All the patients visited our hospital due to persistent swelling and pain in the knee joint. In Case 1, a 6-year-old girl complained of right knee swelling and pain for one month. Case 2 involved a 7-year-old boy with a three-year history of repeated and aggravated left knee swelling and pain. In Case 3, a 2-year-old girl presented with a two-month history of swelling and pain in the right knee. They had no history of recurrent or persistent fever, rash, fatigue, losing weight, and other symptoms of the three patients. And they had no significant past medical history and family history.

**Table 1:** Clinical data of the three pediatric cases with rice body synovitis.

Case No.	Sex	Age (year)	Joint of RBS	Duration of disease	Blood test	Diagnose	Post-operative medication	Follow up period	Reappear
1	Female	6	Right knee	One month	Negative	Congenital synovial hondromatosis	No medication	Three years	No
2	Male	7	Left knee	Three years	T-SPOT positive	Tuberculosis suspected	INZ + EMB	Six months	No
3	Female	2	Right knee	Two months	ANA 1:1,000 positive	JIA with ANA positive	Etanercept + Methotrexate	Six months	No

**Figure 1:**
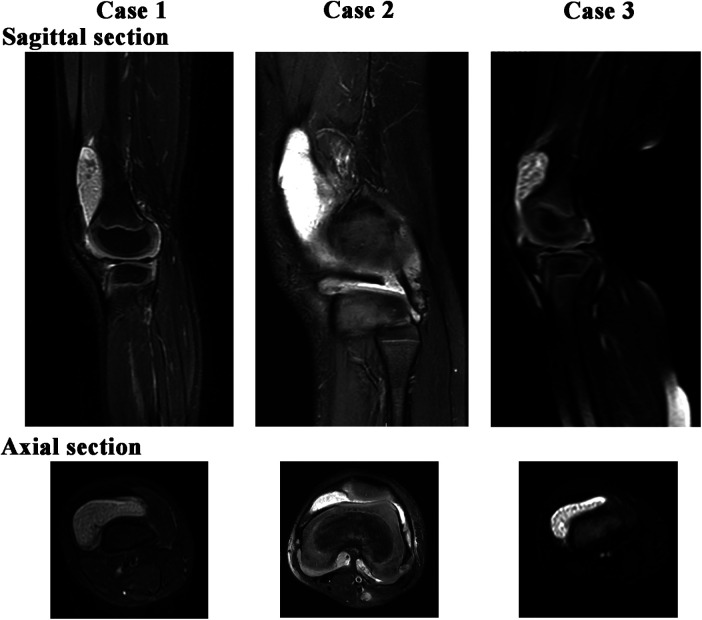
Sagittal and axial MRT2 scanning of knee joint of the three RBS cases. Multiple spot-like high-density spots can be visualized around the area.

**Figure 2:**
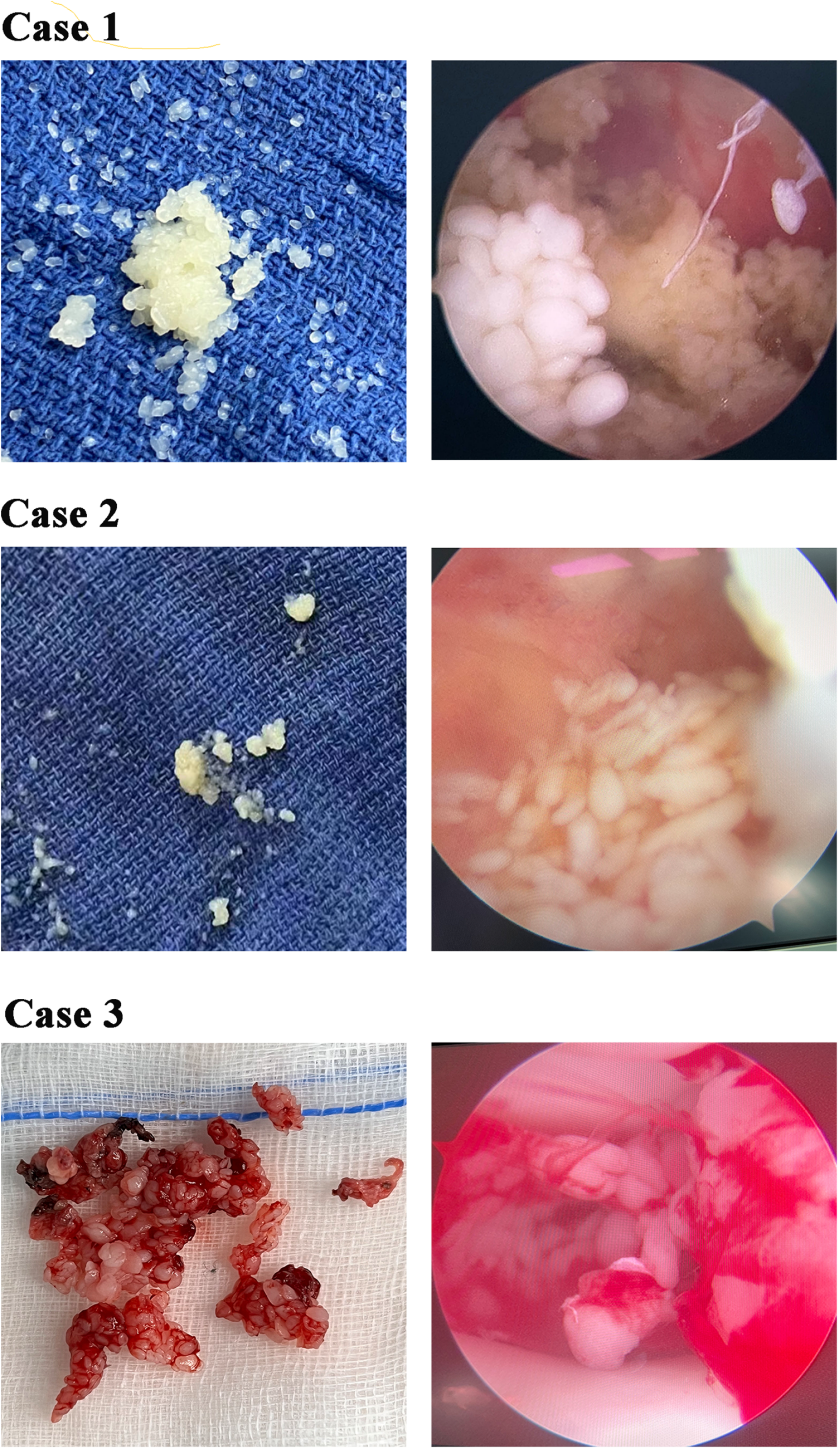
Photograph of multiple rice bodies (RB) that found in the affected knee joint intraoperatively. Left-side figures of every case displayed RB that taken from the affected articular cavity and right-side figures of every case showed the RB form in arthroscopy.

**Figure 3:**
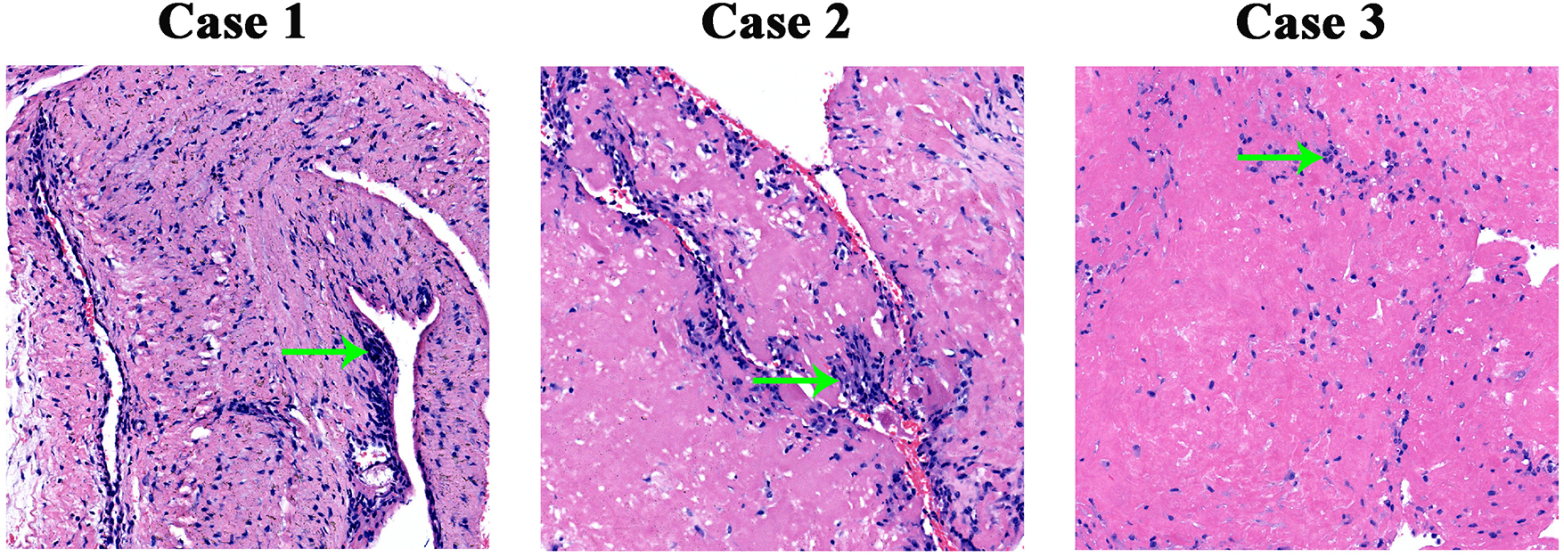
Pathological section of rice body. Every photomicrograph showed the hyperplasia of interstitial vascular and it was infiltrated with inflammatory cells (green arrow), H&E, ×10.

We detected some common clinical features in all three cases. On physical examination, the affected joint showed swelling, tenderness, and heat. However, joint function and range of motion were normal. Each case underwent imaging examinations, including ultrasound and digital radiography and magnetic resonance imaging (MRI) of the affected knee joint. Figure 1 displayed MRI finding of affected joint which significantly suggestive of arthro-synovitis. After a multidisciplinary treatment, the patients were introduced to surgical treatment; a lot of RBS were found in the affected joint (Figure 2). Intraoperative samples were collected and sent for histopathological examination. Figure 3 shows the histopathological findings of the three cases. Hyperplasia of interstitial vasculature and infiltration of inflammatory cells were easily seen microscopically.

Laboratory tests including blood count, erythrocyte sedimentation rate, C-reactive protein, serum muscle enzyme, liver and renal function, electrolyte, routine urine and stool test, rheumatoid factor, HLA-B27, ANA, ENA, anti-cyclic citrullinated protein antibody, humoral and cell immunity, and pathogens especially the T-SPOT were checked. Case 1 showed a normal laboratory examination. Case 2 showed a positive T-SPOT, while ANA 1:1,000 was positive in Case 3. Other laboratory data were normal both in case 2 and case 3. After excluding other causes, the original causes of the three cases were therefore considered to be congenital synovial osteochondroma (Case 1), suspected tuberculosis (Case 2), and ANA-positive JIA (Case 3). In Case 1, the patient received no treatment after surgery; the joint symptom and sign did not show up after a three-year follow-up. In Case 2, the patient was prescribed INZ and EMB for treating tuberculosis; in Case 3, the patient was administered etanercept and methotrexate for JIA treatment. Further, Cases 2 and 3 showed no relapse after a 6-month follow-up.

## 3 Discussion

Reports have shown that knee and shoulder joints are the most commonly involved sites in RBS. Recurrent pain and swelling of the affected joint are the main complaints, with no limitation in joint motion in most reported cases and the present cases. However, some researchers reported that recurrent and long-term inflammation limited articular activity (5, 6). RBS is an unusual complication of chronic bursitis rather than a disease. Multiple cases were reported in the setting of tuberculosis and rheumatoid arthritis; few cases were correlated with pigmented villonodular synovitis, gout, sarcoidosis, or infection (7–10). We searched relative references with the keywords: “rice body”, “bursitis” and “synovitis” in PubMed, yielding 39 references. Table 2 provides a detailed summary of the relative references, which may help us to better understand this disease. In the present study, we report RBS in three children diagnosed with congenital synovial chondromatosis, tuberculosis (unconfirmed), and ANA-positive JIA.

**Table 2:** References review of rice body synovitis.

	Diagnosis	Cases	Age (year)	Sex (F/M)	Duration of symptoms before diagnosis	Location	Surgery	Medication	Follow up	Relapse
Adult	RA	5	28–72	5/0	6–60m	Shoulder (4/5) Wrist (1/5)	Yes (4/5) No (1/5)	Yes (1/5) Not mentioned (4/5)	12m	No
	SLE	2	37–43	2/0	24m	Shoulder (1/2) Finger (1/2)	Yes	Yes	Not mentioned	Not mentioned
	TB	4	41–79	3/1	2–12m	Shoulder (2/4) Hip (1/4) Hand (1/4)	Yes	Yes (3/4) Not mentioned (1/4)	9–12m	No
	Osteoarthritis	4	61–88	3/1	Not mentioned	Knee	Yes	Not mentioned	Not mentioned	Not mentioned
	Tenosynovitis and bursitis	13	50–71	6/7	5–13m	Wrist (4/13) Hand (9/13)	Yes	Yes (3/13) Not mentioned (1/13)	5–12m	No (3/4) Yes (1/4)
	Others	6	31–69	4/2	24–108m	Shoulder (2/6) Knee (1/6) Hand (2/6) Finger (1/6)	Yes	Not mentioned	24m	No
Child	JIA	8	2–11	4/4	2–7m	Knee (7/8) Shoulder (1/8)	Yes	Yes (3/8) Not mentioned (5/8)	6–30m	No
	Tenosynovitis	1	9	0/1	Not mentioned	Tibialis tendon sheath	Yes	Not mentioned	Not mentioned	Not mentioned

RA, rheumatic arthritis; SLE, systemic lupus erythematosus; TB, tuberculosis; JIA, jejunoileal arthropathy.

Considering the same clinical characteristics, the diagnosis and appropriate treatment are always delayed. Thus, blood tests, imaging, and histological examination need to be carried out timely. Ultrasound imaging examination can detect histological microarchitecture of synovial tissues and easily find the rice bodies that floating inside the intra-articular effusion. In clinical practice, it becomes a vital imaging modality in assessing the musculoskeletal systems, especially in pediatrics considering its large availability and the lack of ionizing radiations (11). MRI is the first priority for RBS, which usually shows hypointensity on T1-weighted imaging, hyperintensity on T2-weighted imaging, and iso- or slightly hyperintensity on T1- and T2-weighted imaging when compared to skeletal muscle (12). It appears as a soft tissue mass, debris, blood, or viscous fluid when it is small and resembles ring-enhancing loose bodies when the body is large (13). Some reports indicated that the formation of RB is just an unusual complication of chronic bursitis and is not related to the severity of the imaging findings of arthritis; however, further evidence is needed.

A variety of cells, including phagocytic cells, type C synoviocytes, chondrocytes, and inflammatory cells were found in pathological tissues of RB, which implies that it may have multiple origins, depending on their location. The mechanism of RB formation remains unclear. There are some theories (14–16): microinfarctions after intraarticular synovial inflammation and ischemia; reform and progressive enlargement by fibrin; and alteration in fluid viscosity and fibrinogen content of the synovial fluid. Synovial chondromatosis results from synovial proliferative metaplasia. It rarely evolves into synovial lined bursae and mineralized. The metaplastic cartilage is usually frond like and may produce loose bodies. There are some blue-gray cartilaginous nodules with a diameter of 1–2 cm or larger on the synovial surface of the joint, which can block joint movement in osteochondroma cases. The cartilaginous nodules are composed of synovial connective tissue and are embedded in hyaline chondrocytes under the microscope (5). If a calcified mass is detected in the clinic, the differential diagnosis of synovial chondromatosis, including pigmented villonodular synovitis and tumoral calcinosis due to scleroderma or Sjögren syndrome, must be excluded. Tuberculosis-related RBS is often accompanied by local inflammatory findings, such as local pain, lymphadenitis, and a local progression involving pulmonary tissues, skin, tendons, or bone (17). Caseous necrosis and epithelioid granulomas observed under the microscope are the most helpful findings in diagnosing tuberculosis. Pathological changes in rheumatoid synovitis include regional synovial cell necrosis and erosions covered with cellulose-like deposits. Microscopically, synovial congestion, edema, and infiltration of masses of monocytes, plasma cells, and lymphocytes are easily observed.

There is no consensus when it comes to the therapeutic options of RBS. Either surgery, open arthrotomy, or arthroscopy can be selected to remove RB. However, surgery does not aim for a radical excision and usually cannot prevent recurrence; some patients will relapse after several months or years (7, 18). The RB is a complication of chronic inflammation and other diseases. Thus, strengthening the treatment of the primary disease and enhancing the control of disease activity seem to be more essential and effective in preventing recurrence. However, no standard treatment has been established for RBS. Pharmacotherapy usually depends on the primary disease and its progression. Yamamoto (19) reported that one year of drug therapy after surgery was associated with no relapse in patients with non-tuberculous mycobacterium or fungal infections. Further, the symptoms of the present cases have been controlled over a follow-up period of six months to three years. A further study on which drug should be chosen or how long it should be administered needs to be carried out, considering the adverse drug reactions with long-term therapy. Moreover, a prolonged follow-up is necessary to fully recognize the treatment efficiency and the absence or recurrence of RBS.

## 4 Conclusion

In conclusion, we have reported the clinical characteristics of RBS in three pediatric cases with different primary diseases. Some local symptoms and signs, MRI findings, and pathological features are similar. Treating the primary disease is the key to preventing the recurrence of RBS because surgery only removes the already formed RB. The recurrence and prognosis are still unknown; long-term follow-up is necessary. We hope that these findings may provide a reference for physicians to develop better approaches for the diagnosis and treatment of RBS.

## Data Availability

The original contributions presented in the study are included in the article/Supplementary Material, further inquiries can be directed to the corresponding authors.
